# Draft genome sequence of *Fermentimonas caenicola* strain SIT8, isolated from the human gut

**DOI:** 10.1186/s40793-018-0310-6

**Published:** 2018-04-11

**Authors:** Mamadou Beye, Sofiane Bakour, Sory Ibrahima Traore, Jaishriram Rathored, Noémie Labas, Didier Raoult, Pierre-Edouard Fournier

**Affiliations:** 1 Aix-Marseille Université, URMITE, UM63, CNRS7278, IRD198, Inserm1095, Assistance Publique-Hôpitaux de Marseille, Institut Hospitalo-Universitaire Méditerranée-infection, 19-21 Bd Jean Moulin, 13385 Marseille, cedex 5 France; 2 Special Infectious Agents Unit, King Fahd Medical Research Center, King Abdul Aziz University, Jeddah, Saudi Arabia

**Keywords:** *Fermentimonas caenicola*, Genome, Human gut, Microbiota

## Abstract

We report the properties of a draft genome sequence of the bacterium *Fermentimonas caenicola* strain SIT8 (= CSUR P1560). This strain, whose genome is described here, was isolated from the fecal flora of a healthy 28-month-old Senegalese boy. Strain SIT8 is a facultatively anaerobic Gram-negative bacillus. Here we describe the features of this organism, together with the complete genome sequence and annotation. The 2,824,451-bp long (1 chromosome but no plasmid) contains 2354 protein-coding and 46 RNA genes, including four rRNA genes.

**Electronic supplementary material:**

The online version of this article (10.1186/s40793-018-0310-6) contains supplementary material, which is available to authorized users.

## Introduction

*Fermentimonas caenicola* strain SIT8 (= CSUR P1560) was isolated from the stool of a healthy 28-month-old Senegalese boy as part of a study aiming at cultivating all species within the human gastro-intestinal microbiota. It is a Gram-negative, facultatively anaerobic, indole-negative bacillus. Initially, we had named this bacterium “*Lascolabacillus massiliensis*” as it exhibited unique features among members of the family *Porphyromonadaceae* [1]. However, concomitantly to our work, Hahnke et al. formally described the genus *Fermentimonas* in 2016 [2]. To date, this genus contains only one species, *F. caenicola* [2], the type strain of which, ING2-ESB^T^, exhibits a 100% 16S rRNA sequence identity with strain SIT8. As a consequence, strain SIT8 belongs to the species *F. caenicola*. Strain ING2-ESB2^T^ was isolated from a mesophilic laboratory-scale biogas reactor [2]. To the best of our knowledge, we report here the first isolation of *F. caenicola* from the fecal flora of a human being [3].

Herein, we present a set of features for *F. caenicola* strain SIT8 together with the description of the complete genomic sequence and annotation.

## Organism information

### Classification and features

*Fermentimonas caenicola* strain SIT8 was isolated from the stool of a healthy 28-month-old Senegalese boy (Table 1). The patient’s parents gave informed signed consent, and the agreement of the National Ethics Committee of Senegal and the ethics committee of the IFR48 (Marseille, France, agreement numbers 11–017 and 09–022) were obtained. Strain SIT8 was initially grown after 10 days of culture in a medium enriched with 5% sheep blood and sterile-filtered sheep rumen, in an aerobic atmosphere at 37 °C. The bacterium was sub-cultured on 5% sheep blood-enriched Columbia agar (bioMérieux, Marcy l’Etoile, France) and grew in 24 h at 37 °C in both aerobic and anaerobic conditions.

**Table 1:** Classification and general features of *Fermentimonas caenicola* strain SIT8

MIGS ID	Property	Term	Evidence code^a^
	Classification	Domain *Bacteria*	TAS [21]
		Phylum *Bacteroidetes*	TAS [22, 23]
		Class *Bacteroidia*	TAS [22, 24]
		Order *Bacteroidales*	TAS [22, 25]
		Family *Porphyromonadaceae*	TAS [1, 22]
		Genus *Fermentimonas*	TAS [2, 3]
		Species *Fermentimonas caenicola*	TAS [2, 3]
	Strain	SIT8	IDA
	Gram stain	Negative	IDA
	Cell shape	Bacillus	IDA
	Motility	Not motile	IDA
	Sporulation	Not spore forming	IDA
	Temperature range	Mesophile	IDA
	Optimum temperature	37 °C	IDA
	pH range	6.5–8.5;	IDA
	Carbon source	Galactose, Cellobiose, Lactose, Trehalose, Melezitose, Gentiobiose, Turanose	IDA
GS-6	Habitat	Human gut	IDA
MIGS-6.3	Salinity	0–5 g/L NaCl (w/v)	IDA
MIGS-22	Oxygen requirement	Facultative anaerobe	IDA
MIGS-15	Biotic relationship	Free-living	IDA
MIGS-14	Pathogenicity	Unknown	IDA
MIGS-4	Geographic location	Senegal	IDA
MIGS-5	Sample collection		IDA
MIGS-4.1	Latitude	13.7167	IDA
MIGS-4.2	Longitude	−16.4167	IDA
MIGS-4.4	Altitude	51 m above sea level	IDA

^a^Evidence codes - IDA: Inferred from Direct Assay; TAS: Traceable Author Statement (i.e., a direct report exists in the literature); NAS: Non-traceable Author Statement (i.e., not directly observed for the living, isolated sample, but based on a generally accepted property for the species, or anecdotal evidence). These evidence codes are from the Gene Ontology project [26]. If the evidence is IDA, then the property was directly observed for a live isolate by one of the authors or an expert mentioned in the acknowledgements

**Fig. 1:**
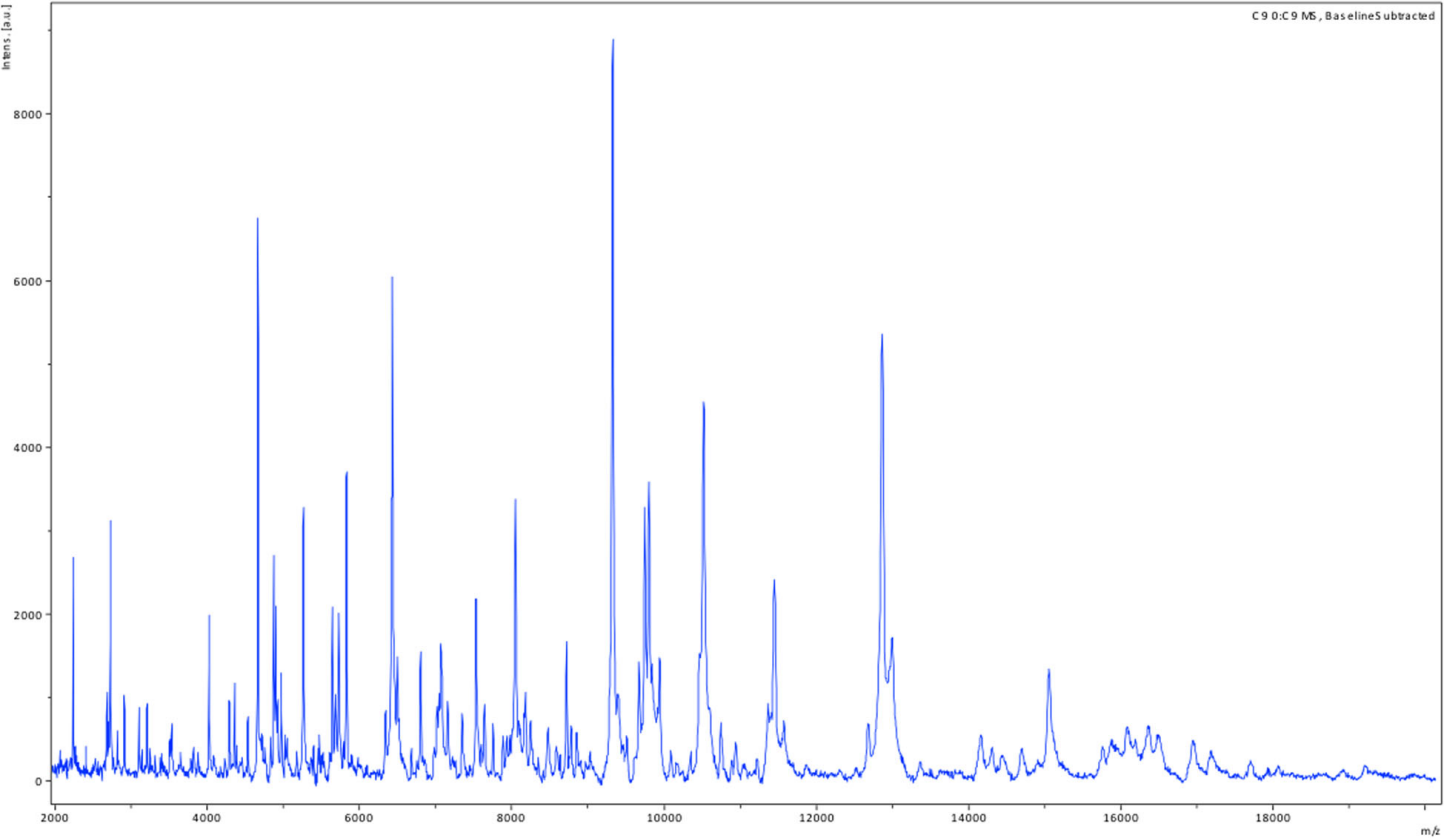
Reference mass spectrum from *Fermentimonas caenicola* SIT8. This reference spectrum was generated by comparison of 12 individual colonies

**Fig. 2:**
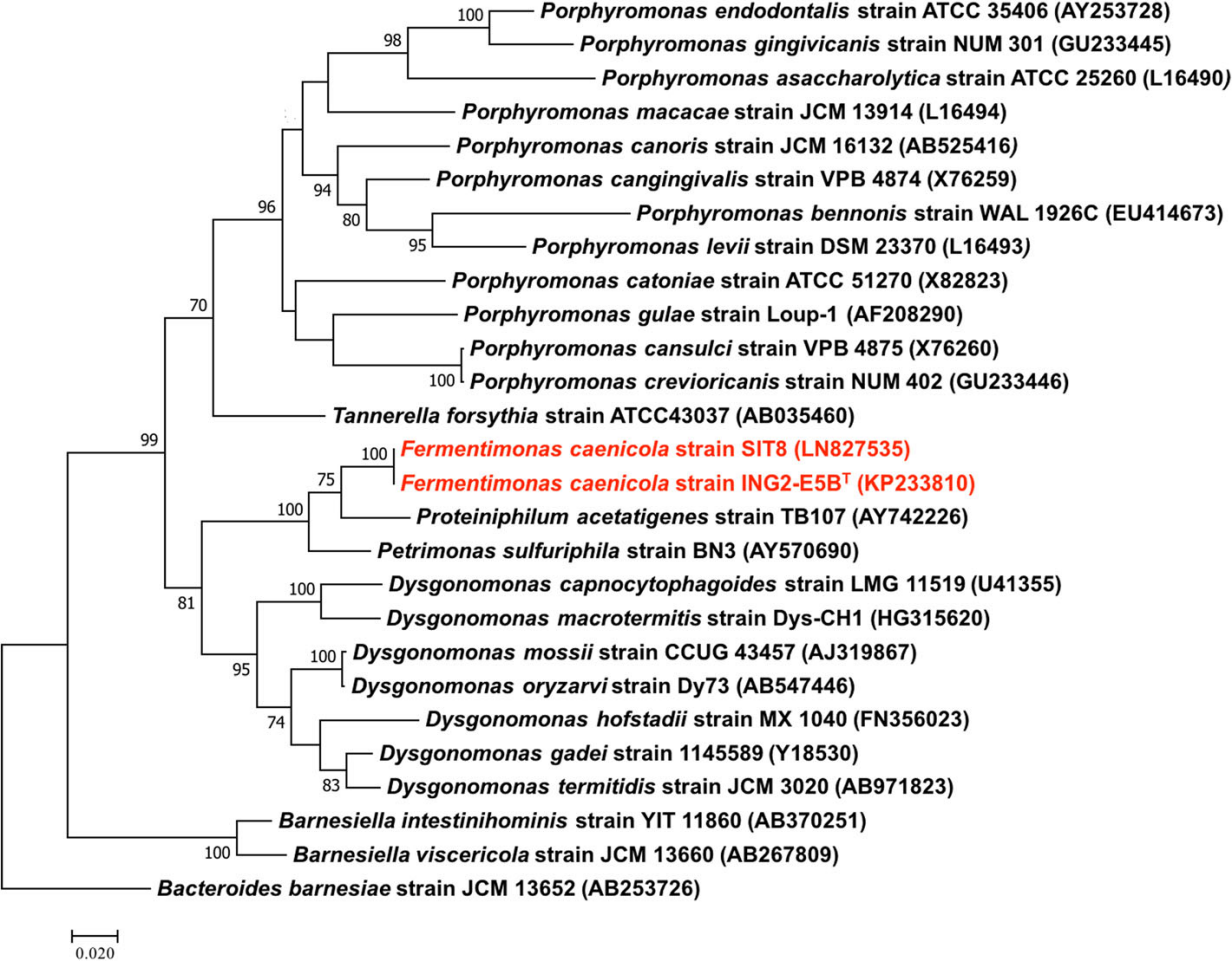
Phylogenetic tree showing the position of *Fermentimonas caenicola* strains SIT8 and ING2-E5B^T^ (red) relative to other phylogenetically close members of the family *Porphyromonadaceae*. GenBank Accession numbers are indicated in parentheses. Sequences were aligned using MUSCLE, and phylogenetic inferences were obtained using the maximum-likelihood method within the MEGA software [20]. Numbers at the nodes are percentages of bootstrap values obtained by repeating the analysis 1000 times to generate a majority consensus tree. Only values ≥ 70% were displayed. The scale bar indicates a 2% nucleotide sequence divergence

Using our systematic matrix-assisted laser desorption-ionization time-of-flight screening on a MicroFlex spectrometer (Bruker Daltonics, Bremen, Germany) [4], strain SIT8 exhibited no significant score, suggesting that it was not a member of any known species (Fig. 1). We added the spectrum from strain SIT8 to our database (Fig. 1). Strain SIT8 exhibited a 100% 16S rRNA sequence identity with Fermentimonas caenicola strain ING2-E5B^T^ (GenBank accession KP233810), the phylogenetically closest species with a validly published name in nomenclature (Fig. 2). The 16S rRNA sequence of strain SIT8 has been deposited in GenBank under number LN827535.

Growth at different temperatures (29, 37 and 55 °C) was tested. Growth of the strain was tested in 5% sheep blood-enriched Columbia agar (bioMérieux) and Tryptic Soy agar (Becton–Dickinson, Le Pont-de-Claix, France) under anaerobic and microaerophilic conditions using the GENbag anaer and GENbag microaer systems, respectively (bioMérieux), and under aerobic conditions, with or without 5% CO_2_. Growth was tested for salt tolerance, with 0–5, 50 and 100% (*w*/*v*) NaCl. The pH range for growth was tested at pH 6.5 and 8.5 using Tryptic Soy agar. Phenotypic tests were performed using API ZYM, API 20NE and API 50CH strips (bioMérieux). In vitro susceptibility to antibiotics was determined using the disk-diffusion method on 5% sheep blood-enriched Mueller–Hinton agar (bioMérieux).

Electron microscopy was performed with detection Formvar coated grids which were deposited on a 40 μL bacterial suspension drop and incubated at 37 °C for 30 min, followed by a 10 s incubation on ammonium molybdate 1%. Grids were then observed using a Morgagni 268D transmission electron microscope (Philips) at an operating voltage of 60 kV.

**Fig. 3:**
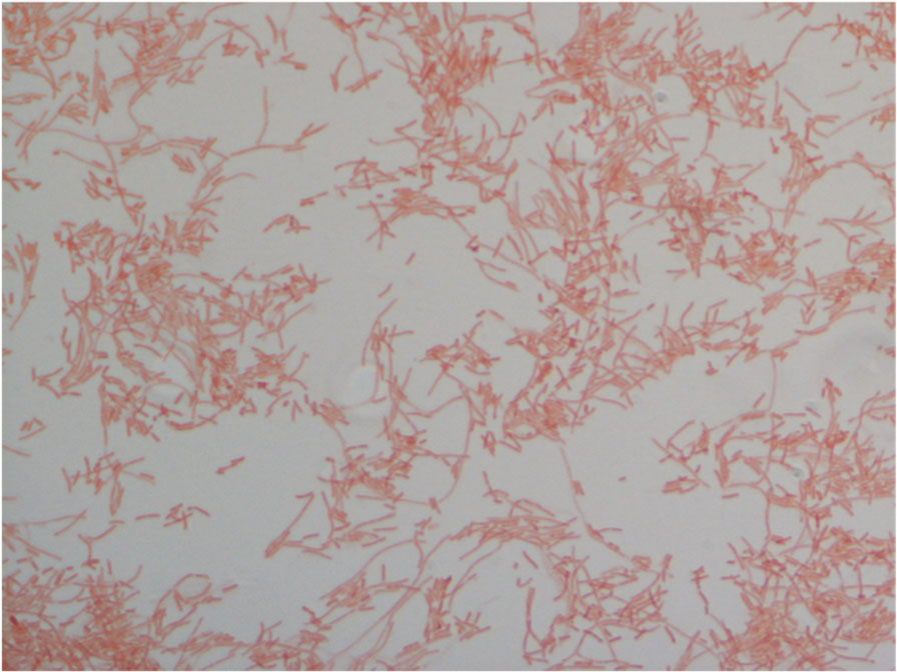
Gram staining of *Fermentimonas caenicola* strain SIT8

**Fig. 4:**
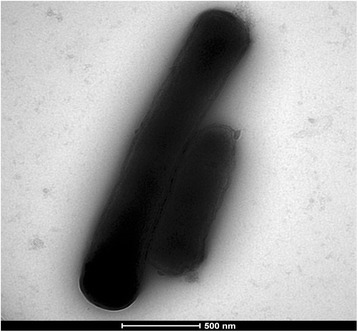
Transmission electron microscopy of *Fermentimonas caenicola* strain SIT8 using a Morgani 268D microscope (Philips, Amsterdam, The Netherlands) at an operating voltage of 60 kV. Scale bar = 500 nm

Different growth temperatures (29 °C, 37 °C, 55 °C), pH and salinity were determined. Growth was obtained at 29 and 37 °C, with optimal growth at 37 °C, at pH 6.5–8.5 and at NaCl concentration of 0 to 5 g/L. Strain growth was observed in both aerobic and anaerobic conditions and with or without 5% CO_2_. Colonies were pale grey and 1.5 mm in diameter on 5% sheep blood-enriched Columbia agar (bioMérieux). A motility test was negative. Cells were Gram-negative, rod-shaped, polymorphic (Fig. 3), unable to form spores and exhibited a mean diameter of 0.35 μm (range 0.3–0.4 μm) and a mean length of 3.8 μm (range 1–8.8 μm) (Fig. 4).

Strain SIT8 exhibited neither catalase nor oxidase activities. Using an API ZYM strip (bioMérieux), positive reactions were observed for alkaline phosphatase, acid phosphatase, and N-acetyl-β-glucosaminidase. Negative reactions were noted for esterase, esterase-lipase, lipase, leucine arylamidase, β-glucosidase, β-galactosidase, α-mannosidase, α-fucosidase, cystine arylamidase, valine arylamidase, trypsin, α-chymotrypsin, α-glucosidase, α-galactosidase, β-glucuronidase, and Naphthol-AS-BI-phosphohydrolase*.*

Using an API 50 CH strip (bioMérieux), positive reactions were observed after 48 h of incubation for the fermentation of D-arabinose, D-galactose, D-glucose, D-mannose, N-acetylglucosamine, amygdalin, arbutin, salicin, D-cellobiose, D-maltose, D-lactose, D-trehalose, D-melezitose, amidon, glycogen, gentiobiose, D-turanose, and potassium-5-ketogluconate. Negative reactions were observed for the fermentation of glycerol, erythritol, L-arabinose, D-ribose, D-xylose, L-xylose, D-adonitol, methyl-β-D-xylopyranoside, D-fructose, L-sorbose, L-rhamnose, dulcitol, inositol, D-mannitol, D-sorbitol, methyl-αD-xylopyranoside, methyl-αD-glucopyranoside, D-mellibiose, D-saccharose, inulin, D-raffinose, xylitol, D-lyxose, D-tagatose, D-fucose, L-fucose, D-arabitol, L-arabitol, potassium gluconate, potassium 2-ketogluconate.

Using an API 20NE strip (bioMérieux), a positive reaction was obtained only for esculin hydrolysis while negative reactions were observed for nitrate reduction, urease, indole production, arginine dihydrolase, glucose fermentation, arabinose, mannose, mannitol, N-acetyl-glucosamine, maltose, gluconate, caprate, adipate, malate, citrate, phenyl-acetate assimilation, and gelatin hydrolysis.

Strain SIT8 was susceptible to penicillin, amoxicillin, amoxicillin/clavulanic acid, ticarcillin, ceftriaxone, cefalotin, imipenem, gentamicin, trimethoprim/sulfamethoxazole, erythromycin, doxycycline, metronidazole, vancomycin, rifampicin, ciprofloxacin, nitrofurantoin, and colistin, but resistant to kanamycin.

#### Chemotaxonomic data

Cellular fatty acid methyl ester analysis was performed by GC/MS. Two samples were prepared with approximately 30 mg of bacterial biomass per tube harvested from several culture plates. Fatty acid methyl esters were prepared as described by Sasser [5]. GC/MS analyses were carried out as described before [6]. Briefly, fatty acid methyl esters were separated using an Elite 5-MS column and monitored by mass spectrometry (Clarus 500 - SQ 8 S, Perkin Elmer, Courtaboeuf, France). Spectral database search was performed using MS Search 2.0 operated with the Standard Reference Database 1A (NIST, Gaithersburg, USA) and the FAMEs mass spectral database (Wiley, Chichester, UK).

Hexadecanoic acid is the most abundant fatty acid (45%). 9-Octadecenoic acid and 9,12-Octadecadienoic acid are also abundant unsaturated fatty acids (23 and 20% respectively) (Additional file 1: Table S1).

## Genome sequencing information

### Genome project history

The strain was selected for sequencing on the basis its 16S rRNA similarity, phylogenetic position, and phenotypic differences with the other members of the family *Porphyromonadaceae*, and is part of a culturomics study of the human microbiome. It is the second published genome from the *F. caenicola* species. Table 2 shows the project information and its association with MIGS version 2.0 compliance [7]. The genome Genbank accession number is CTEJ01000000. The genome consists of 2 scaffolds.

**Table 2:** Project information

MIGS ID	Property	Term
MIGS 31	Finishing quality	High-quality draft
MIGS-28	Libraries used	Mate-paire 250 bp library
MIGS 29	Sequencing platforms	Illumina Miseq
MIGS 31.2	Fold coverage	272
MIGS 30	Assemblers	Spades
MIGS 32	Gene calling method	Prodigal
	Locus Tag	Not indicated
	Genbank ID	CTEJ01000000
	Genbank Date of Release	AUGUST 04,2015
	GOLD ID	Not indicated
	BIOPROJECT	
MIGS 13	Source Material Identifier	CSUR P1560
	Project relevance	Study of human gut

### Growth conditions and DNA preparation

Strain SIT8 (CSUR P1560) was sub-cultured on 5% sheep blood-enriched Columbia agar (bioMérieux) and grew in 24 h at 37 °C in anaerobic atmosphere. Eight Petri dishes were harvested and resuspended in 4x100μl of G2 buffer (EZ1 DNA Tissue kit, Qiagen). A first mechanical lysis was performed by glass powder on the Fastprep-24 device (MP Biomedicals, Santa Ana, California, USA) using 2 × 20 seconds cycles. DNA was then treated with 2.5 μg/μL lysozyme (30 min at 37 °C) and extracted using the BioRobot EZ 1 Advanced XL (Qiagen). DNA was then concentrated and purified with the Qiamp kit (Qiagen). DNA concentration was 70.7 ng/μl as determined by the Genios Tecan fluorometer, using the Quant-it Picogreen kit (Invitrogen).

### Genome sequencing and assembly

The genomic DNA of *F. caenicola* strain SIT8 was sequenced on a MiSeq sequencer (Illumina Inc., San Diego, CA, USA) with the Mate-Pair strategy. The gDNA was barcoded in order to be mixed with 9 other projects with the Nextera Mate-Pair sample prep kit (Illumina).

The gDNA was quantified by a Qubit assay with the high sensitivity kit (Life technologies, Carlsbad, CA, USA) to 82.6 ng/μl. The Mate-Pair library was prepared with 1.5 μg of gDNA using the Nextera mate pair Illumina guide. The gDNA was simultaneously fragmented and tagged with a Mate-Pair junction adapter. The fragmentation pattern was validated on an Agilent 2100 BioAnalyzer (Agilent Technologies, Santa Clara, CA, USA) with a DNA 7500 labchip. DNA fragments ranged in size from 1.5 kb up to 11 kb with an optimal size at 4.33 kb. No size selection was performed and 662 ng of tagmented fragments were circularized. The circularized DNA was mechanically sheared to small fragments with an optimal at 1200 bp on the Covaris device S2 in T6 tubes (Covaris, Woburn, MA, USA). The library profile was visualized on a High Sensitivity Bioanalyzer LabChip (Agilent Technologies) and the final concentration library was measured at 61.4 nmol/l.

The libraries were normalized at 2 nM and pooled. After a denaturation step and dilution at 15 pM, the pool of libraries was loaded. Automated cluster generation and sequencing run were performed in a single 39-h run in a 2 × 251-bp.

Total information of 7.84 Gb was obtained from an 884 K/mm^2^ cluster density with a cluster passing quality control filters of 92.7% (15,478,025 passing filter paired reads). Within this run, the index representation for *F. caenicola* strain SIT8 was determined to be 13.25%. The 2,050,529 paired reads were trimmed and then assembled in 2 scaffolds.

### Genome annotation

Open Reading Frames were predicted using Prodigal [8] with default parameters but the predicted ORFs were excluded if they were spanning a sequencing gap region. The predicted bacterial protein sequences were searched against the GenBank database [9] and the Clusters of Orthologous Groups databases [10] using BLASTP. The tRNAScanSE tool [11] was used to find tRNA genes, whereas ribosomal RNAs were found by using RNAmmer [12] and BLASTn against the GenBank database. Signal peptides and numbers of transmembrane helices were predicted using SignalP [13] and TMHMM [14] respectively. ORFans were identified if their BLASTP E-value was lower than 1e-03 for alignment lengths greater than 80 amino acids. If alignment lengths were smaller than 80 amino acids, we used an E-value of 1e-05. Such parameter thresholds have already been used in previous works to define ORFans. Artemis [15] was used for data management, and DNA Plotter [16] was used for visualization of genomic features. The Mauve alignment tool was used for multiple genomic sequence alignment [17]. To identify putative orthologues and estimate the pan/core-genome composition, comparative genomic analysis was carried out between the two *F. caenicola* strains SIT8 and ING2-E5B^T^ using bidirectional Best Blast from the BLASTClust algotithm [18], and then specific genes were checked by tBLASTN. We estimated the mean level of nucleotide sequence similarity at the genome level using the digital DNA-DNA hybridization and the genome-to-genome distance calculator Web server as previously reported [19].

## Genome properties

**Fig. 5:**
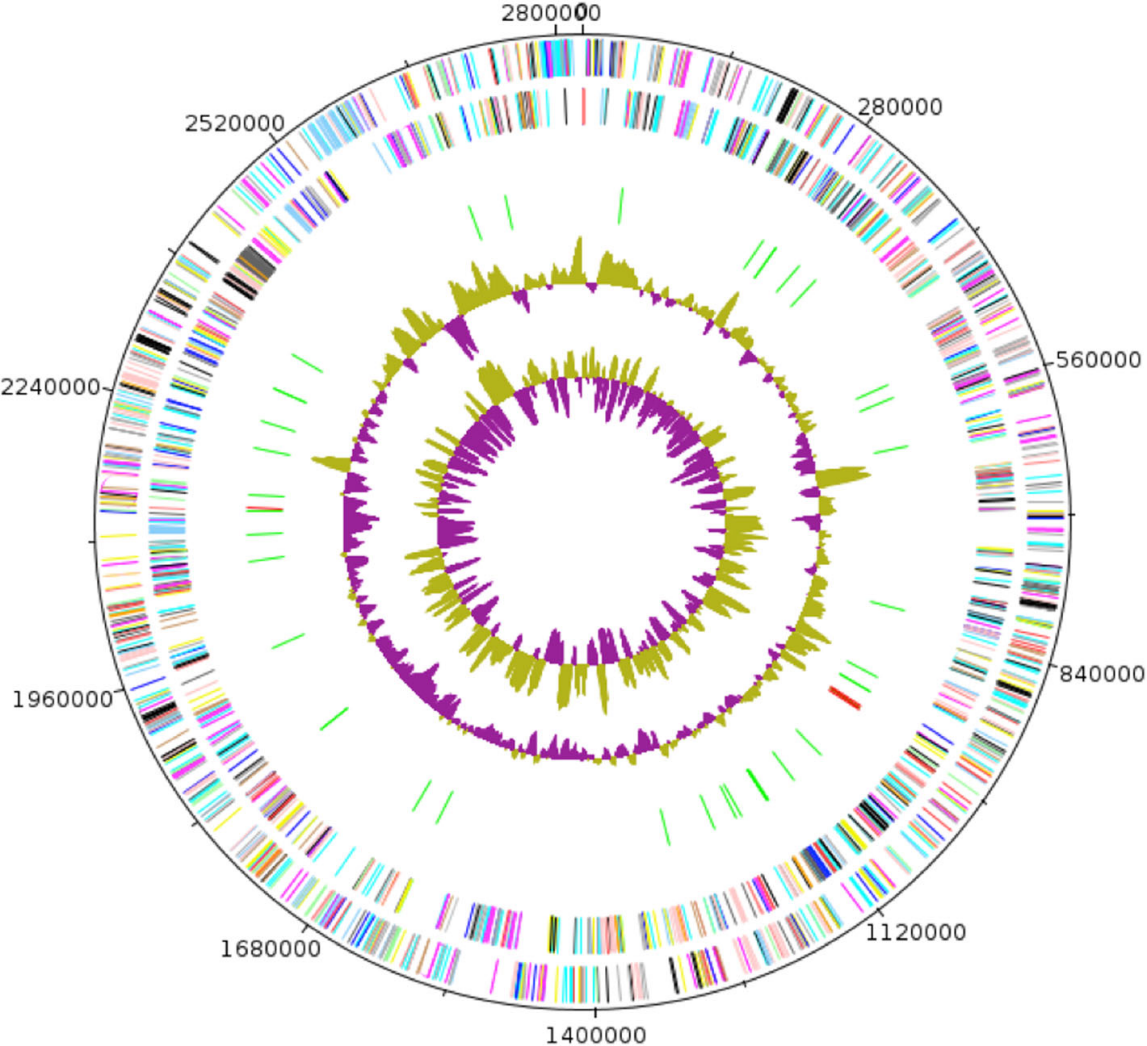
Graphical circular map of the chromosome of *Fermentimonas caenicola* strain SIT8. From the outside in, the outer two circles show open reading frames oriented in the forward (coloured by COG categories) and reverse (coloured by COG categories) directions, respectively. The third circle marks the rRNA gene operon (red) and tRNA genes (green). The fourth circle shows the GC % content plot. The inner-most circle shows GC skew, purple indicating negative values and olive for positive values

The genome of strain SIT8 is 2,824,451-bp long with a 37% G + C content (Table 3; Fig. 5). Of the 2400 predicted genes, 2354 are protein-coding genes, and 46 encode rRNAs. Four rRNA genes (one 16SrRNA, one 23S rRNA and two 5S rRNA) and 42 predicted tRNA genes were identified in the genome. A total of 1668 genes (69.5%) were assigned a putative function. Twenty-eight genes were identified as ORFans (1.7%). The remaining genes were annotated as hypothetical proteins (732 genes, 30.5%). The properties and the statistics of the genome are summarized in Table 3.

**Table 3:** Genome statistics

Attribute	Strain SIT8		Strain ING2-E5B^T^	
	Value	% of total	Value	% of total
Genome size (bp)	2,824,451	100%	2,808,926	100%
DNA coding (bp)	2,599,342	92.03%	2,582,364	91.93%
DNA G + C (bp)	1,045,046	37%	1,047,729	37.3%
DNA scaffolds	2		1	
Total genes	2400	100%	2455	100%
Protein coding genes	2354	98.08%	2405	97.96%
RNA genes	46	1.92%	50	2.04%
Genes with function prediction	1668	69.5%	1758	71.61%
Genes assigned to COGs	1693	70.54%	1705	69.45%
Genes with Pfam domains	2221	92,54%	2184	88.96%
Genes with signal peptides	657	27.37%	502	20.44%
Genes with transmembrane helices	437	18.21%	539	21.96%
ORFans genes	28	1.17%	22	0.89%
CRISPR repeats	0		1

The distribution of genes into COGs functional categories is presented in Table 4.

**Table 4:** Number of genes associated with the 25 general COG functional categories

Code	Strain SIT8		Strain ING2-E5B^T^		Description
	Value	% of total	Value	% of total	
J	133	5.65	135	5.61	Translation
A	0	0	0	0	RNA processing and modification
K	85	3.61	83	3.45	Transcription
L	90	3.82	130	5.41	Replication, recombination and repair
B	0	0	0	0	Chromatin structure and dynamics
D	20	0.85	21	0.87	Cell cycle control, mitosis and meiosis
Y	0	0	0	0	Nuclear structure
V	29	1.23	29	1.21	Defense mechanisms
T	45	1.91	40	1.66	Signal transduction mechanisms
M	146	6.20	125	5.20	Cell wall/membrane biogenesis
N	2	0.09	2	0.08	Cell motility
Z	0	0	0	0	Cytoskeleton
W	0	0	0	0	Extracellular structures
U	17	0.72	17	0.70	Intracellular trafficking and secretion
O	69	2.93	69	2.87	Posttranslational modification, protein turnover, chaperones
C	123	5.23	121	5.03	Energy production and conversion
G	142	6.03	143	5.95	Carbohydrate transport and metabolism
E	144	6.12	143	5.95	Amino acid transport and metabolism
F	54	2.29	55	2.29	Nucleotide transport and metabolism
H	77	3.27	76	3.16	Coenzyme transport and metabolism
I	57	2.42	58	2.41	Lipid transport and metabolism
P	118	5.01	106	4.41	Inorganic ion transport and metabolism
Q	11	0.47	13	0.54	Secondary metabolites biosynthesis, transport and catabolism
R	216	9.18	213	8.86	General function prediction only
S	115	4.89	126	5.24	Function unknown
–	661	28.08	700	29.1	Not in COGs

*COGs* Clusters of Orthologous Groups database

## Insights from the genome sequence

To date, one genome from the *Fermentimonas* genus has been published. Here, we compared the genome sequence of *F. caenicola* strains SIT8 (Genbank accession number CTEJ01000000) and ING2-E5B^T^ (Genbank accession number NZ_LN515532).

The draft genome of strain SIT8 (2.87 Mb) has a larger size than that of strain ING2-E5B^T^ (2.85 Mb). The G + C content of strains SIT8 and ING2-E5B^T^ are comparable (37% vs 37.3%, respectively). The gene content of strain SIT8 is lower than that of strain ING2-E5B^T^ (2400 vs 2455, respectively). The ratio of genes per Mb of strain SIT8 is lower than that of strain ING2-E5B^T^ (836 vs 861, respectively).

The distribution of genes into COGs functional categories is comparable between strains SIT8 and ING2-E5B^T^ (Table 4). The genomic comparison identified a pangenome of 2681 genes and core genome of 2096 genes. Strains SIT8 and ING2-E5B^T^ harboured 273 and and 312 specific genes, respectively. Functional annotation of the unique genes from strain SIT8 revealed that 48.35% are found into COGs functional categories against 52.56% for strain ING2-E5B^T^ (Additional file 1: Table S2). The COG functional classification of the specific genes from strain SIT8 showed that 10.62% play a role in cell wall, membrane biogenesis and 6.59% in inorganic ion transport and metabolism (Additional file 1: Table S2). In contrast, 16.99% of specific genes from strain ING2-E5B^T^ are involved in replication, recombination and repair and 6.73% in carbohydrate transport and metabolism (Additional file 1: Table S2).

Strains SIT8 and ING2-E5B^T^ share a mean 95.5% dDDH value.

## Conclusions

We describe the phenotypic, phylogenetic and genomic characteristics of *Fermentimonas caenicola* strain SIT8. This bacterial strain was isolated from a stool specimen of a healthy 28-month-old Senegalese boy. Strain SIT8 (= CSUR P1560) is the first *F. caenicola* strain isolated from humans.

## Additional file

Additional file 1: **Table S1.** Fatty acid composition of *Fermentimonas caenicola* strain SIT8. **Table S2.** Number of specific genes associated with the 25 general COG functional categories. (DOCX 59 kb)

## Abbreviations

bp: base pairs
COG: Clusters of Orthologous Groups
CSUR: Collection de Souches de l’Unité des Rickettsies
DDH: DNA-DNA hybridization
FAME: Fatty Acid Methyl Ester
GC/MS: Gas Chromatography/Mass Spectrometry
GGDC: Genome-to-Genome Distance Calculator
kb: kilobases
MALDI-TOF MS: Matrix-assisted laser-desorption/ionization time-of-flight mass spectrometry
ORF: Open Reading Frame
URMITE: Unité de Recherche sur les Maladies Infectieuses et Tropicales Emergentes
