# Supplementary material for: Draft genome sequence of Fermentimonas caenicola strain SIT8, isolated from the human gut
Source: Stand Genomic Sci. 2018 Apr 11;13:8. doi: 10.1186/s40793-018-0310-6 (PMC5896035; doi:10.1186/s40793-018-0310-6)
Supplement: Supplementary file 1 — Table S1. Fatty acid composition of Fermentimonas caenicola strain SIT8. Table S2. Number of specific genes associated with the 25 general COG functional categories. (DOCX 59 kb) [file 40793_2018_310_MOESM1_ESM.docx]

**Table S1**: Fatty acid composition of *Fermentimonas caenicola* strain SIT8

| **Fatty acids** | **Name** | **Mean relative % (a)** |
| --- | --- | --- |
| 16:0 | Hexadecanoic acid | 45.4 ± 4.1 |
| 18:1n9 | 9-Octadecenoic acid | 22.9 ± 1.6 |
| 18:2n6 | 9,12-Octadecadienoic acid | 19.9 ± 2.7 |
| 14:0 | Tetradecanoic acid | 6.0 ± 0.5 |
| 15:0 | Pentadecanoic acid | 1.5 ± 0.3 |
| 18:0 | Octadecanoic acid | 1.5 ± 0.2 |
| 12:0 | Dodecanoic acid | 1.0 ± 0.1 |
| 18:1n7 | 11-Octadecenoic acid | TR |
| 10:0 | Decanoic acid | TR |
| 16:1n9 | 7-Hexadecenoic acid | TR |
| 15:0 iso | 13-methyl-tetradecanoic acid | TR |
| 15:0 anteiso | 12-methyl-tetradecanoic acid | TR |

a Mean peak area percentage ; TR = trace amounts < 1 %

**Table S2**: Number of specific genes associated with the 25 general COG functional categories

| Code | Strain SIT8 | |  | **Strain ING2-E5B^T^** | | Description |
| --- | --- | --- | --- | --- | --- | --- |
|  | Value | **% of total^a^** |  | Value | **% of total^a^** |  |
| J | 0 | 0 |  | 2 | 0.64 | Translation |
| A | 0 | 0 |  | 0 | 0 | RNA processing and modification |
| K | 7 | 2.56 |  | 7 | 2.25 | Transcription |
| L | 7 | 2.56 |  | 53 | 16.99 | Replication, recombination and repair |
| B | 0 | 0 |  | 0 | 0 | Chromatin structure and dynamics |
| D | 0 | 0 |  | 1 | 0.32 | Cell cycle control, mitosis and meiosis |
| Y | 0 | 0 |  | 0 | 0 | Nuclear structure |
| V | 6 | 2.2 |  | 6 | 1.92 | Defense mechanisms |
| T | 7 | 2.56 |  | 2 | 0.64 | Signal transduction mechanisms |
| M | 29 | 10.62 |  | 8 | 2.56 | Cell wall/membrane biogenesis |
| N | 1 | 0.37 |  | 1 | 0.32 | Cell motility |
| Z | 0 | 0 |  | 0 | 0 | Cytoskeleton |
| W | 0 | 0 |  | 0 | 0 | Extracellular structures |
| U | 1 | 0.37 |  | 1 | 0.32 | Intracellular trafficking and secretion |
| O | 5 | 1.83 |  | 5 | 1.6 | Posttranslational modification, protein turnover, chaperones |
| C | 6 | 2.20 |  | 4 | 1.28 | Energy production and conversion |
| G | 13 | 4.76 |  | 21 | 6.73 | Carbohydrate transport and metabolism |
| E | 2 | 0.73 |  | 2 | 0.64 | Amino acid transport and metabolism |
| F | 1 | 0.37 |  | 0 | 0 | Nucleotide transport and metabolism |
| H | 1 | 0.37 |  | 1 | 0.32 | Coenzyme transport and metabolism |
| I | 1 | 0.37 |  | 3 | 0.96 | Lipid transport and metabolism |
| P | 18 | 6.59 |  | 7 | 2.25 | Inorganic ion transport and metabolism |
| Q | 0 | 0 |  | 2 | 0.64 | Secondary metabolites biosynthesis, transport and catabolism |
| R | 18 | 6.59 |  | 16 | 5.13 | General function prediction only |
| S | 9 | 3.3 |  | 22 | 7.05 | Function unknown |
| - | 141 | 51.65 |  | 148 | 47.44 | Not in COGs |

COGs, Clusters of Orthologous Groups database

Total specific genes of strain SIT8 = 273

Total specific genes of strain ING2-E5B^T^ = 313
